# Development of a high-throughput assay to detect antibody inhibition of low pH induced conformational changes of influenza virus hemagglutinin

**DOI:** 10.1371/journal.pone.0199683

**Published:** 2018-06-27

**Authors:** Jessica F. Trost, Elizabeth H. LeMasters, Feng Liu, Paul Carney, Xiuhua Lu, Kanetsu Sugawara, Seiji Hongo, James Stevens, David A. Steinhauer, Terrence Tumpey, Jacqueline M. Katz, Min Z. Levine, Zhu-Nan Li

**Affiliations:** 1 Influenza Division, Centers for Disease Control and Prevention, Atlanta, Georgia, United States of America; 2 Department of Microbiology and Immunology, Emory University School of Medicine, Atlanta, Georgia, United States of America; 3 Department of Infectious Diseases, Yamagata University Faculty of Medicine, Yamagata, Japan; Icahn School of Medicine at Mount Sinai, UNITED STATES

## Abstract

Many broadly neutralizing antibodies (bnAbs) bind to conserved areas of the hemagglutinin (HA) stalk region and can inhibit the low pH induced HA conformational changes necessary for viral membrane fusion activity. We developed and evaluated a high-throughput virus-free and cell-free ELISA based low pH induced HA Conformational Change Inhibition Antibody Detection Assay (HCCIA) and a complementary proteinase susceptibility assay. Human serum samples (n = 150) were tested by HCCIA using H3 recombinant HA. Optical density (OD) ratios of mAb HC31 at pH 4.8 to pH 7.0 ranged from 0.87 to 0.09. Our results demonstrated that low pH induced HA conformational change inhibition antibodies (CCI) neutralized multiple H3 strains after removal of head-binding antibodies. The results suggest that HCCIA can be utilized to detect and characterize CCI in sera, that are potentially broadly neutralizing, and serves as a useful tool for evaluating universal vaccine candidates targeting the HA stalk.

## Introduction

Infections caused by influenza A viruses are a major public health concern, place extraordinary burdens on health care systems and local, national, and global economies. Over the past century, only a small subset of the 18 antigenically distinct hemagglutinin (HA) subtypes and 11 neuraminidase (NA) subtypes circulating in nature have acquired the capacity to establish sustained transmission in the human population [1–3]. The H1N1, H2N2, and H3N2 subtype viruses continued to evolve following their emergence as a pandemic virus, circulating for varying periods of time among humans [4, 5]. Seasonal influenza A and B viruses require year-round surveillance to ascertain whether genetic changes resulting in antigenic drift occurred; antigenic changes necessitate regular updating of seasonal influenza vaccine viruses [6]. Another public health challenge posed by influenza viruses is the emergence of novel influenza A viruses from animal reservoirs. Over the past 20 years, viruses of the H5, H6, H7, H9, and H10 subtypes have resulted in thousands of zoonotic infections [7]. While none of these subtypes have yet acquired the ability to transmit efficiently among humans, annual zoonotic epidemics such as the yearly A(H7N9) virus epidemics occurring in China since 2013 have resulted in nearly 1,500 laboratory-confirmed human infection cases, highlighting their pandemic potential [8]. Clearly, the threat of an emerging novel HA subtype virus from animal reservoirs remains at the forefront of pandemic preparedness efforts.

Current vaccines targeting seasonal and potential pandemic influenza viruses focus on stimulating strain-specific neutralizing antibodies that largely recognize epitopes on the globular head of the HA molecule and egg-based platforms are used to produce them [6, 9]. This strategy provides seasonal influenza vaccines that are only modestly effective when vaccines are well matched with circulating strains, and substantially less effective when poorly matched with a newly emerged antigenically drifted virus [10, 11]. Furthermore, most current vaccine technologies provide limited flexibility to respond rapidly to the emergence of pandemic influenza viruses or antigenically drifted viruses. As such, improved vaccines and therapies are needed to better prepare for the emergence of an antigenically drifted seasonal influenza virus or a novel influenza virus for which the human population does not currently possess immunity. Though the definition of “universal influenza vaccine” is under debate, several conserved viral antigens including HA, NA, the extracellular domain of Matrix 2 (M2e) protein, and Nucleoprotein (NP) are targeted for “universal” or broadly protective vaccines that are evaluated in pre-clinical or clinical trials [3, 12]. Stimulation of broadly neutralizing antibodies (bnAbs) targeting conserved epitopes of the major viral glycoprotein, HA, have been the focus for the development of next generation influenza vaccines [3, 12]. Broadly neutralizing monoclonal antibodies (bn mAbs) have shown either cross influenza: type (A and B); influenza A virus HA group; HA subtype; or HA strains neutralizing activity, additionally some of them recognize the HA receptor-binding site (RBS) and HA vestigial esterase subdomain. BnAbs neutralize influenza virus by multiple mechanisms such as blocking virus attachment to cells, inhibiting low pH induced HA conformational changes related to membrane fusion activity, inhibiting virus release, and Fc receptor related functions [3, 13–20]. Many mouse and human bn mAbs that bind to more conserved epitopes in HA stalk domain have been isolated from hybridoma, phage display libraries, and single cell PCR techniques [3, 21–25]. Most HA stalk-binding bn mAbs recognize conformational rather than linear epitopes [5, 25] and while they may act through different functions, to date, all have included a mechanism which prevented the HA from undergoing the low pH induced conformational changes necessary to mediate viral and endosomal membrane fusion, either by blocking proteolytic cleavage of HA0 or locking the HA in its pre-fusion, metastable conformation [26, 27].

There are substantial ongoing efforts to design and develop immunogens that elicit strong antibody responses against the conserved HA stalk region, including designing “headless” HA immunogens with or without stabilization, the mini-stem approach [28–31], HA stem nanoparticles [32], a hyperglycosylated HA globular head domain [33], elimination of the N-linked glycosylation sites in the HA stalk domain [34], and utilization of chimeric HA and chimeric viruses [5, 30].

As these new vaccine technologies are developed and undergo pre-clinical and clinical evaluation [1, 5, 9, 35], appropriate assays and immune correlates of protection will be needed to evaluate the protective efficacy of these countermeasures. Various assays have been used to evaluate the mechanism of action and protection conferred by low pH induced HA conformational change inhibition antibodies (CCI), including heterokaryon formation inhibition [5, 25, 26], red blood cell (RBC) lysis [15], proteinase susceptibility [36–38], and animal model studies [5, 32, 36, 39]. While these approaches can readily detect bn mAbs, they are not suitable for detecting bnAbs in human serum samples or discriminating the bnAbs acting through inhibition of low pH induced HA conformational changes associated with membrane fusion activity from Abs that inhibit hemagglutination activity. Given the need for better assays to enrich characterization of CCI that bind to the conserved HA stalk domain and possess broadly neutralizing activity, we sought to develop and validate a novel assay to contribute to bridging this gap. To date, all reported HA stalk-binding bnAbs can inhibit low pH induced HA conformational changes; therefore, we hypothesize that detection of CCI can be used to evaluate fusion inhibition antibodies that are potentially broadly neutralizing especially after HA head-binding antibodies are removed via serum adsorption with the globular head domain HA1 (GH HA1).

In the present manuscript, we focused on the development of a novel high-throughput virus-free and cell-free ELISA-based assay to detect CCI. This new assay detects CCI that are fusion inhibiting and potentially broadly neutralizing. Importantly, both low pH induced HA Conformational Change Inhibition Antibody Detection Assay (HCCIA) and the complementary proteinase susceptibility assay are capable of being adapted for high-throughput with the utilization of Pierce Nickel Coated Clear 96-Well Plates (nickel-coated plates) (ThermoFisher Scientific, IL). Furthermore, we have demonstrated that HCCIA results are consistent with conventional proteinase susceptibility and neutralization titers.

## Materials and methods

### Source of serum samples

From 2000 to 2008, 150 human serum samples were collected from anonymous U.S. residents who were born between 1932 and 1985. The use of these sera was approved by Centers for Disease Control Institutional Review Board (CDC Approval # 1652). Initially, a convenient human serum pool (Pool) was also used in this study, the pool consisted of a mixture of equal volume of anonymous sera collected from 25 U.S. residents in 2010. These anonymous sera were provided by a contract organization (Accelovance, Inc. MD). The use of these sera were exempt from the review of CDC’s Institutional Review Board.

### Expression and purification of recombinant HA

**Fig 1:**
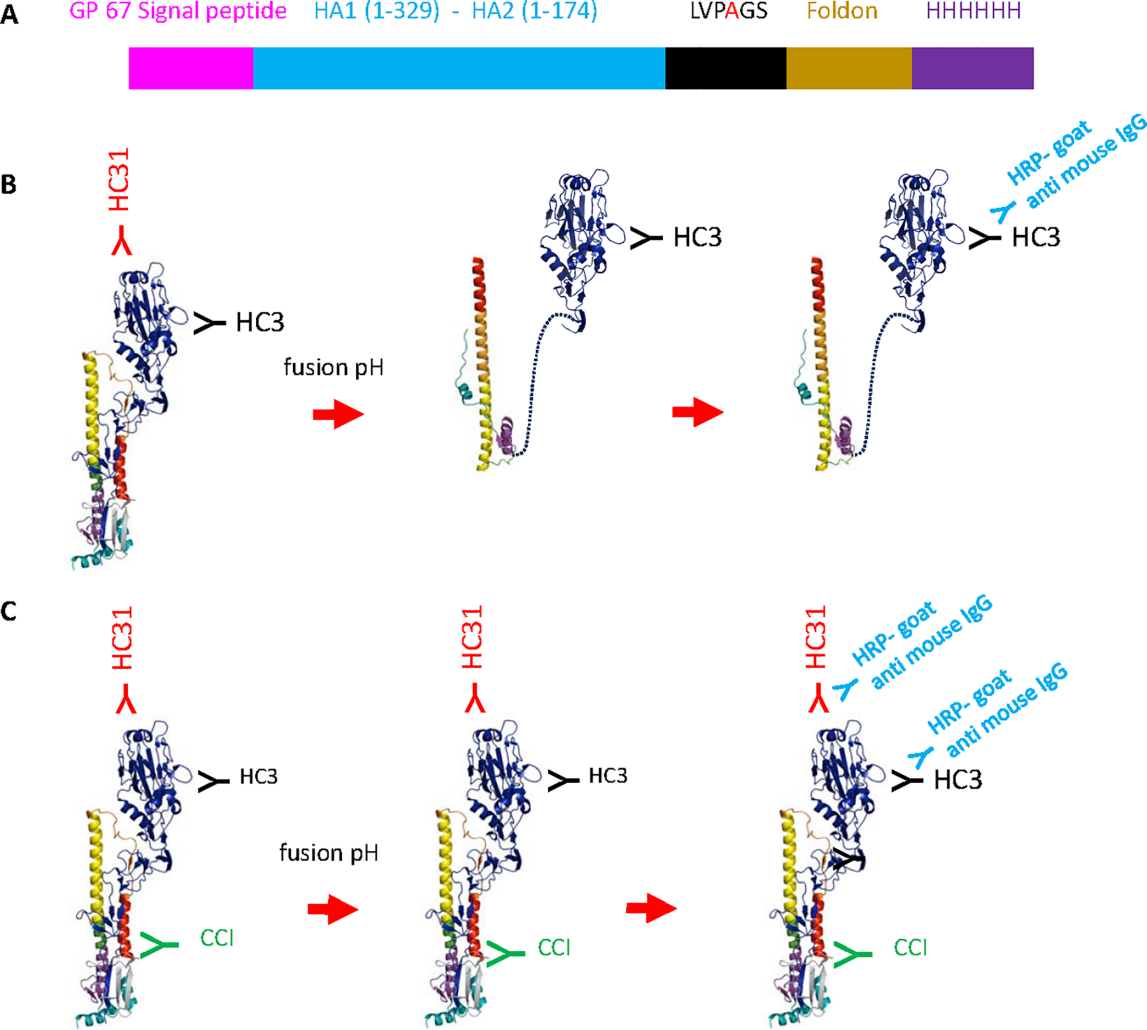
Principle of HCCIA. **A.** The linear schematic of the rHA as described [41] with some modifications, not to scale. N-terminal baculovirus GP67 signal peptide, HA ectodomain, mutated thrombin cleavage site from LVPRGS to LVPAGS, foldon, and 6 histidine-Tag. **B.** The structure shown in the schematic was modified from a published HA structure (PDB No. 1HTM and 4WE4) [41–43]. Though trimeric rHA was used in the HCCIA, the H3 rHA is shown as a monomer in Fig 1. In the absence of CCI, H3 rHA would undergo conformational changes at the pH of fusion and HC31 (HC67 for H3 rHA, or 1/87 mAb for H2 rHA) would lose the ability to bind to the rHA, while HC3 (shown in black) would bind to the H3 rHA when in its fusogenic conformation. **C.** In the presence of CCI (shown in green), H3 rHA would remain “locked” in its metastable pre-fusion conformation and maintain recognition by both HC3 (shown in black) and HC31 (shown in red), as well as HC67 or 1/87 mAb for H2 rHA.

Recombinant HAs (rHAs) from A/swine/Missouri/2124514/2006 (H2N3) and A/Hong Kong/1/68 (H3N2) which is antigenically similar to HA from A/Aichi/2/68 (H3N2) were selected to represent antigenically distinct group 1 and 2 HAs, respectively. cDNAs corresponding to baculovirus GP67 signal peptide, HA ectodomain (HA1 residues from 1 to 329 and HA2 residues from 1 to 174) followed by a mutated thrombin cleavage site (from LVPRGS to LVPAGS), T4 fibritin foldon sequence (foldon), and six histidine-Tag to aid in purification were expressed from recombinant baculovirus-infected insect cells (Fig 1A) [40, 41]. Purity, trimerization, and receptor binding activity were confirmed as described previously [40, 41].

### Mouse monoclonal antibodies

Mouse anti-H2 HA (A/Japan/305/57-like) mAbs C179 (Clontech Laboratories, Inc. CA), 1/87 and 2/9 described in our previous study [44], and mouse anti-H3 HA (A/Aichi/2/68) mAbs (HC3, HC31, HC67, HC100, HC159, and HC263) were characterized in previous studies [25, 44–46]. The pH sensitive mouse anti-HA mAbs: anti-H2 HA (H2N2) 1/87 and anti-H3 HA (H3N2) (HC31 and HC67) were used to determine binding activities to various pH-treated H2 and H3 rHAs, respectively.

### Confirmation of H3 rHA folding by ELISA using mouse monoclonal antibodies

Ectodomain (in-house made) and GH HA1 (Sino Biological, Inc. China) of rHAs from A/Hong Kong/1/68 (H3N2) were coated on nickel-coated plates and an ELISA was performed with rabbit anti-H3N2 (A/Aichi/2/68) sera and a panel of mouse anti-H3 HA (A/Aichi/2/68) conformation specific mAbs HC3, HC31, HC67, HC100, and HC263, as described previously [46, 47].

### Determination of the optimal TPCK-treated trypsin concentration for cleavage of rHA bound to nickel-coated plates

To determine the N-tosyl-L-phenylalanyl chloromethyl ketone (TPCK)-treated trypsin (trypsin, Sigma, MO) concentration to cleave rHA, rHA was bound to nickel-coated plates and subjected to digestion with two-fold serially diluted trypsin starting from 16,000 ng/ml to 32 ng/ml in PBS and PBS only conditions at 37°C for 15 minutes. The digestion mixture was removed and washed once with PBS, then the rHA bound to the 96-well nickel-coated plate was eluted by 1X reducing sample loading buffer (SLB) supplemented with 0.5 M Imidazole (EMD Millipore, MA). Samples were heated at 95°C for 5 minutes and separated on 4–12% Criterion gel (Bio-Rad, CA), followed by Western blot using anti-H2 mouse mAb (2/9) [44] or rabbit anti-H3N2 (A/Aichi/2/68) sera.

### Detection of low pH induced conformational change of rHA bound to nickel-coated plates by ELISA

Nickel-coated plates were coated with 200 ng/well of H2 or H3 rHA at 4°C overnight. The plate was washed once with 100 μl of PBS/well to remove unbound rHA, treated with 100 μl of 200 ng/ml trypsin in PBS at 37°C for 15 minutes to cleave HA0 into HA1 and HA2, and the plate wells were washed twice with 100 μl of 200 ng/ml soybean trypsin inhibitor (Sigma, MO) in PBS to avoid further trypsin digestion following low pH treatment. The plate was incubated with 2% BSA/0.05% Tween 20/PBS for 1 hour and subsequently incubated with pH adjusted buffer (20 mM citrate, 20 mM HEPES, 150 mM NaCl, 2 mM CaCl_2_) in 0.2 unit increments ranging from pH 4.6–5.8 and pH 7.0 at 37°C for 1 minute. The plate was washed once with PBS and incubated for 30 minutes at room temperature with 0.05% glutaraldehyde/PBS to fix rHA on plate. The pH-specific mAbs 1/87 for H2 rHA, and HC31 and HC67 for H3 rHA were added and incubated at room temperature for 1 hour. The plate was washed with 0.05% Tween 20/PBS followed by incubation with an HRP-conjugated secondary antibody (SouthernBiotech, AL) for 1 hour. Fifty microliters of 3,3',5,5'-Tetramethylbenzidine (TMB) (KPL, MD) was added to each well, the reaction was terminated by adding 50 μl of TMB stop solution/well (KPL, MD), and the OD_450 nm_ was measured via spectrophotometer.

### Detection of low pH induced conformational change of rHA bound to nickel-coated plates by a proteinase susceptibility assay

HA at its low pH conformation is more sensitive to proteinase digestion than that at neutral pH conformation, this biochemical property of HA can be exploited in a proteinase susceptibility assay [48]. Briefly, rHA bound to a nickel-coated 96-well plate was treated with 100 μl of 200 ng/ml of trypsin per well, washed twice with 200 ng/ml of soybean trypsin inhibitor, then treated with neutral (pH 7.0) or low (pH 4.8) pH buffer (20 mM citrate, 20 mM HEPES, 150 mM NaCl, 2 mM CaCl_2_) conditions for one minute at 37°C. The buffer was removed by tapping of the plate followed by addition of 50 μl of 0, 0.1, 1, or 10 μg/ml trypsin in PBS at 37°C for 2 hours. In our initial observations, some rHAs were released in the trypsin digestion mixture, since there are three arginine/lysine residues within the foldon sequence of mutated thrombin cleavage site rHA used in this study (S1 Fig). Therefore, without a subsequent washing step, total well samples which included the digestion mixture and rHAs bound to the nickel-coated plate were eluted by adding 50 μl of 2X non-reducing SLB supplemented with 1M imidazole. The samples were separated by SDS-PAGE under non-reducing conditions and probed with anti H2 HA mAb (2/9) or rabbit antisera against A/Aichi/2/68 (H3N2) virus. HA proteins were detected by chemiluminescence (GE Healthcare Bio-Sciences, PA) of a Western blot after incubation with an HRP-conjugated secondary antibody (SouthernBiotech, AL).

### Validation of HCCIA using the bn mAb C179

Mouse mAb C179 binds to the HA stalk domain and neutralizes multiple subtypes of group 1 influenza A viruses by inhibiting the low pH induced conformational changes of HA that are associated with membrane fusion [25, 49]. To validate the HCCIA, the nickel-coated 96-well plate bound by 200 ng/well of H2 rHA was treated with 100 μl of 200 ng/ml of trypsin, incubated in the absence or presence of 100 ng/well C179 (Clontech Laboratories, Inc. CA) in 2% BSA/0.05% Tween 20/PBS for one hour, and treated with pH adjusted buffer (20 mM citrate, 20 mM HEPES, 150 mM NaCl, 2 mM CaCl_2_) in 0.2 unit increments ranging from pH 4.6–5.8 and pH 7.0 at 37°C for one minute. Both C179 and 1/87 are mouse mAbs, and as such, the plate was incubated with 1:50 diluted unlabeled goat anti-mouse IgG (UNLB, Southern Biotech, AL) for one hour to prevent the HRP-conjugated secondary antibody (SouthernBiotech, AL) binding to mAb C179. The plate was fixed with 0.05% glutaraldehyde in PBS followed by one PBS wash. The neutral pH-specific detection mAb 1/87 was used to detect conformation of H2 rHA, as described above.

The proteinase susceptibility assay was performed in the absence or presence of 100 ng/well of C179 followed by a Western blot using mouse anti-H2 HA mAb 2/9 [44] as described above.

### Detection of antibodies in human sera that inhibit low pH induced HA conformational changes associated with membrane fusion activity using HCCIA

The procedure used to detect antibodies in human sera that inhibit low pH induced HA conformational changes associated with membrane fusion activity was similar to that as described above. Briefly, H3 rHA bound to a nickel-coated 96 well plate was incubated with human sera diluted in 2% BSA/0.05% Tween 20/PBS at 1:40, 1:400, or 1:4,000 dilutions for one hour, washed 3 times with 0.05% Tween 20/PBS, treated with neutral (pH 7.0) or low (pH 4.8) pH buffer (20 mM citrate, 20 mM HEPES, 150 mM NaCl, 2 mM CaCl_2_). The plates were fixed with 0.05% glutaraldehyde/PBS followed by addition of pH-specific mAb HC31 and processed as described above. The ratio of the OD_450 nm_ value of mAb HC31 at pH 4.8 to pH 7.0 was calculated from two to four independent assays. The OD value ratio was used as an indication of the presence or absence of antibodies that inhibit low pH induced HA conformational changes necessary for triggering membrane fusion.

To confirm the HCCIA data, a conventional proteinase susceptibility assay was performed after incubation with diluted human sera. Additional steps were performed as described previously, except that rabbit antisera against A/Aichi/2/68 (H3N2) virus was used in the Western blot to reflect appropriate antisera for the H3 rHA.

### Serum adsorption with GH HA1 from A/Hong Kong/1/68 (H3N2) only or followed by adsorption with GH HA1 from A/Perth/16/2009 (H3N2)

The convenient human serum pool (Pool) and the highest positive sample #115 in HCCIA were further analyzed by HI and traditional microneutralization (TMN) assays to explore correlations between HCCIA and existing assays. Many serum samples showed HI titers against the A/Aichi/2/68 (H3N2) and A/Perth/16/2009 (H3N2) viruses, indicating the presence of antibodies that bound to the HA head domain and showed hemagglutination inhibition activity. To focus the detection of stalk-binding antibodies, human serum samples were adsorbed with GH HA1 to remove antibodies against the HA head domain. The adsorption procedure was modified from the methods described by Khurana and colleagues [50]. Briefly, 50 μl of PureProteome™ Nickel Magnetic Bead System (Millipore, MA) was coated with 30 μg of GH HA1 from A/Hong Kong/1/68 (H3N2) (Sino Biological, Beijing, China) or A/Perth/16/2009 (H3N2) for 30 minutes. Antibody-bead conjugates were washed with PBS to remove unbound rHA and 250 μl of 1:5 diluted human serum sample was added and incubated for one hour. The solution was subjected to centrifugation at 300 x g for 5 minutes at 4°C, the adsorbed sera was harvested, treated with 150 μl of Receptor Destroying Enzyme (RDE, Denka Seiken, Japan), and incubated at 37°C overnight. One hundred microliters of PBS was added and the mixture was incubated at 56°C for 30 minutes to inactivate RDE (for a final dilution of 1:10).

### HI and traditional microneutralization assays

HI was performed as described by WHO guidelines [51] accessed on Aug. 10, 2016, serum samples were treated with a receptor destroying enzyme (Denka-Seiken, Tokyo, Japan) to remove nonspecific inhibitors and adsorbed with packed turkey red blood cells (TRBCs) to remove nonspecific agglutinins before testing for the presence of HI antibody by using 4 hemagglutination units (HAU) of virus and 0.5% TRBCs in PBS. The HI titer was determined by the reciprocal of the highest dilution of serum inhibiting virus hemagglutination [51]. The traditional microneutralization (TMN) assay was performed as described [52, 53]. Briefly, serial 2-fold dilutions of the heat inactivated serum samples were mixed with 100 tissue culture infective doses (100 TCID_50_) of virus. The virus-serum mixtures were incubated for 2 hours, then added to an MDCK cell monolayer to detect un-neutralized virus. The plates were incubated in DMEM supplemented with 1 μg/ml TPCK trypsin at 37°C with 5% CO_2_ for 3 days. The presence of un-neutralized viruses were detected by visualizing cytopathic effects (CPE) and hemagglutinating activity. The neutralizing antibody titer was the reciprocal of the highest dilution of serum protecting the MDCK cells against the virus infection.

## Results

In this study, we opted to utilize the H2 and H3 rHAs, as they are representatives of group 1 and group 2 HAs and there are detection mAbs that differentially recognize HA neutral and low pH conformations, such as HC31, HC67, and 1/87. A major goal in the development of HCCIA was to create an *in vitro*, cell-free, virus-free assay that could be performed at BSL-2 conditions. As shown in Fig 1A, H2 and H3 rHAs were expressed by using a baculovirus expression system, the construct that was used to express the rHA proteins included an N-terminal baculovirus GP67 signal peptide, HA ectodomain (HA1 1–329 and HA2 1–174), a mutated thrombin cleavage site, foldon, and a C-terminal six Histidine tag to enable binding to nickel-coated plates at correct HA orientation as would be seen on the viral surface [40, 41]. The histidine tagged rHAs bound to nickel-coated plates are conveniently released by imidazole for proteinase susceptibility assay.

The general principle of the HCCIA is schematically detailed in Fig 1. Briefly, rHA is bound to nickel-coated 96-well plates, cleaved with exogenous trypsin, probed with antibody or serum, and subjected to treatment with pH adjusted buffer to trigger low pH induced HA conformational changes associated with viral membrane fusion activity. In the absence of an Ab that inhibits low pH induced HA conformational changes, the HA undergoes structural rearrangement at the pH of fusion and adopts the highly stable helical rod structure (Fig 1B) [42, 54]. In this case, the low pH conformation rHA only reacts with mAb HC3 for H3 rHA and loses reactivity to Abs specific for the neutral pH structure, such as HC31 for H3 rHA (Fig 1B). Alternatively, in the presence of an Ab that inhibits low pH induced HA conformational changes, HAs will remain “locked” in the pre-fusion metastable conformation and maintain their recognition by neutral pH-specific Ab, such as HC31 (Fig 1C). The HCCIA provides a process to evaluate and select for antibodies that inhibit low pH induced HA conformational changes necessary for viral membrane fusion activity.

### Optimization of rHA precursor (HA0) cleavage activation conditions by trypsin

**Fig 2:**
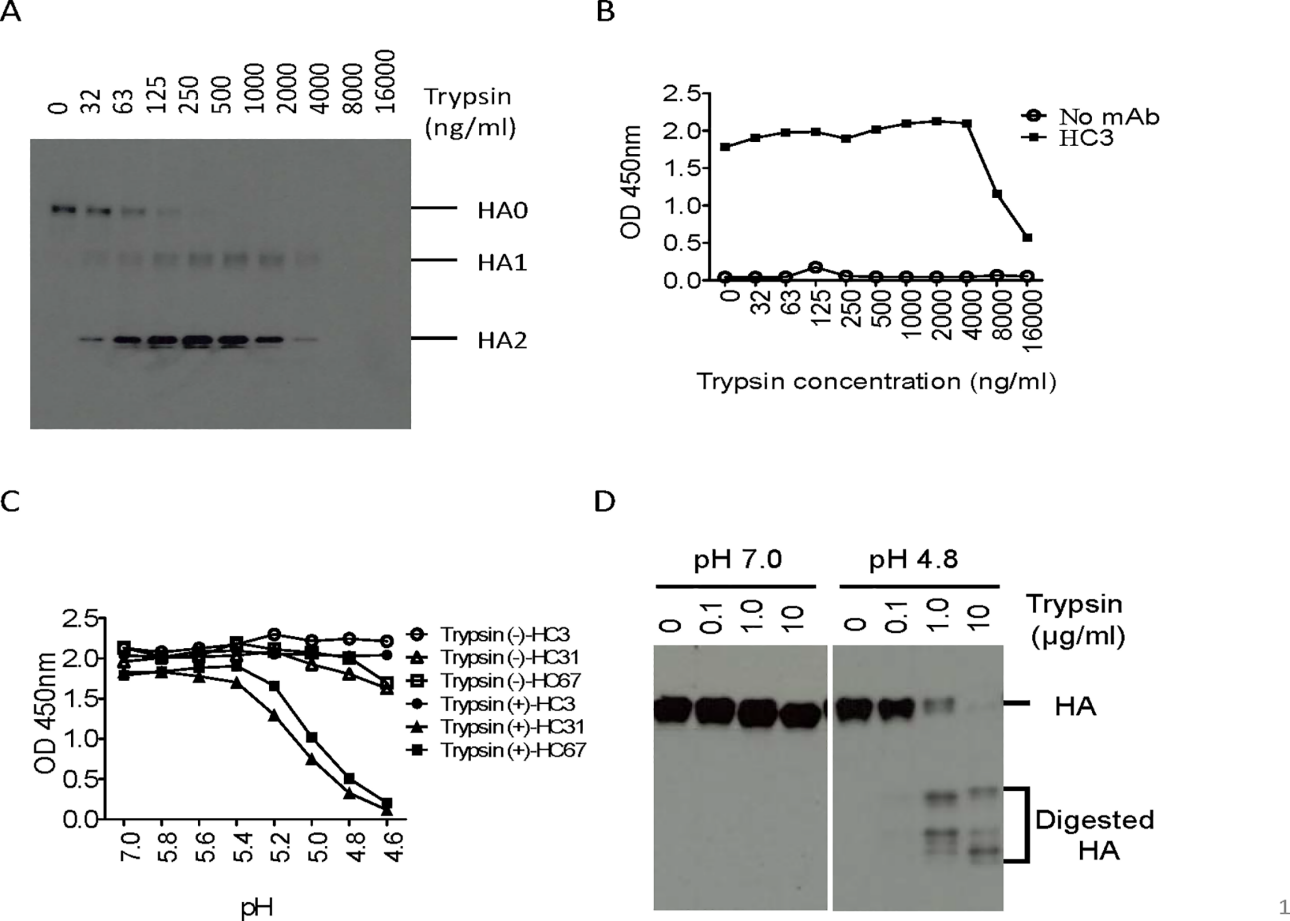
Determination of low pH induced conformational change of H3 rHA on 96-well nickel-coated plate. Optimization of trypsin concentration for cleavage of rHA coated on nickel-coated plates. H3 rHA bound nickel-coated plates were digested with two-fold serially diluted trypsin starting from 16,000 ng/ml to 32 ng/ml in PBS and PBS only as control. **A.** rHAs were eluted by 1X reducing SLB supplemented with 0.5M Imidazole followed by Western blot using anti H3 rabbit sera. **B.** Trypsin treated rHAs were analyzed by ELISA using anti H3 monoclonal antibody HC3. **C.** Cleavage of HA0 into HA1 and HA2 of H3 rHA by trypsin was essential for low pH induced HA conformational changes. H3 rHAs bound to nickel-coated plates were treated with 100 μl of 200 ng/ml trypsin to cleave HA0 into HA1 and HA2. The plate was treated with a series of pH buffers followed by fixation with 0.05% glutaraldehyde in PBS. ELISA was performed by using pH-specific mAbs HC31 and HC67, and HC3 served as a control for H3 rHA. **D.** The proteinase susceptibility assay was performed to confirm the low pH induced HA conformational changes in Fig 2D. The H3 rHA bound nickel-coated plate was treated with pH 7.0 or pH 4.8 followed by 0, 0.1, 1, or 10 μg/ml trypsin digestion. Total sample which included the digestion mixture and rHA remaining bound to the plate were entirely eluted from the nickel-coated plate by adding an equal volume of 2X non-reducing SLB supplemented with 1M imidazole and were separated by SDS-PAGE under non-reducing conditions. PAGE-separated proteins were transferred to a nitrocellulose membrane and probed with a rabbit anti A/Aichi/1/68 (H3N2) antisera. HA proteins were detected by chemiluminescence with an HRP-conjugated secondary antibody.

The H2 and H3 rHA proteins expressed from the baculovirus-infected insect cells are in the uncleaved, HA0 form. As such, it was necessary to initially determine the optimal trypsin concentration for HA cleavage for these assays. Purified H2 and H3 rHAs were immobilized on nickel-coated 96-well plates and treated with varying concentrations of trypsin, ranging from 0–16,000 ng/ml. The results showed that sufficient rHA0 was cleaved into HA1 and HA2 between 125–250 ng/ml (Fig 2A). These data were further confirmed by ELISA using anti-H3 HA (A/Aichi/2/68) mouse mAb HC3 (Fig 2B). Based on these titration experiments, we utilized a concentration of 200 ng/ml of trypsin for HA cleavage activation in all subsequent experiments.

### Detection of low pH induced conformational changes of rHA bound to nickel-coated 96-well plates by ELISA and a proteinase susceptibility assay

To demonstrate that the cleavage of the HA0 into HA1 and HA2 was sufficient to trigger the low pH induced HA conformational changes associated with membrane fusion activity, H3 rHA was immobilized on a nickel-coated 96-well plate, treated with 200 ng/ml trypsin or without exogenous trypsin, followed by exposure to pH adjusted buffers in 0.2 unit increments, ranging from 4.6–5.8 and 7.0. The pH treated rHA was subsequently detected with conformation specific monoclonal antibodies HC3, HC31, or HC67 using an ELISA. The HC3 antibody that recognizes both the neutral and the low pH conformation of HA was used as control, whereas HC31 and HC67 react only with the neutral pH form. In the absence of trypsin, HC31 and HC67 signals were similar following incubation at all pH values tested, as expected, both mAbs showed about 90% OD_450nm_ value at pH 4.6 compared to that at pH 7.0. The similar signal occurred because uncleaved HA0 could not undergo the low pH induced conformational changes (Fig 2C). However, when H3 rHA was treated with 200 ng/ml of trypsin, HC3 bound equally to rHA exposed to neutral and low pH buffers while the pH specific mAbs, HC31 and HC67, showed reduced binding at lower pH treated H3 rHA (Fig 2C). The low pH induced conformational change of H3 rHA was further confirmed using the conventional proteinase susceptibility assay (Fig 2D). The foundation of this assay is that at a neutral pH, cleaved HA is resistant to further digestion by trypsin; however, at the lower pH of fusion, the HA1 subunit of cleaved HA is susceptible to further digestion by various proteases, including trypsin [48]. As shown in Fig 2D, H3 rHA was resistant to exogenous trypsin treatment at pH 7.0, but sensitive at pH 4.8, as evidenced by the faster migrating cleavage products shown in lanes treated with 1.0 and 10 μg/ml of trypsin. The majority, approximately 80%, of rHA following pH 4.8 treatment remained on the nickel-coated plates, compared to neutral pH 7.0 treated samples in the proteinase susceptibility assay. A majority of the rHA remaining on the plate following low pH treatment suggested that the low pH treatment did not significantly interfere with the nickel and histidine tag in this assay (Fig 2D). Taken together, these data demonstrate that the H3 rHA is being cleaved into the di-sulfide linked HA1 and HA2 subunits and is capable of undergoing the necessary conformational changes associated with membrane fusion activity.

### Inhibition of low pH induced conformational change of H2 rHA by C179 was demonstrated by HCCIA and a proteinase susceptibility assay

**Fig 3:**
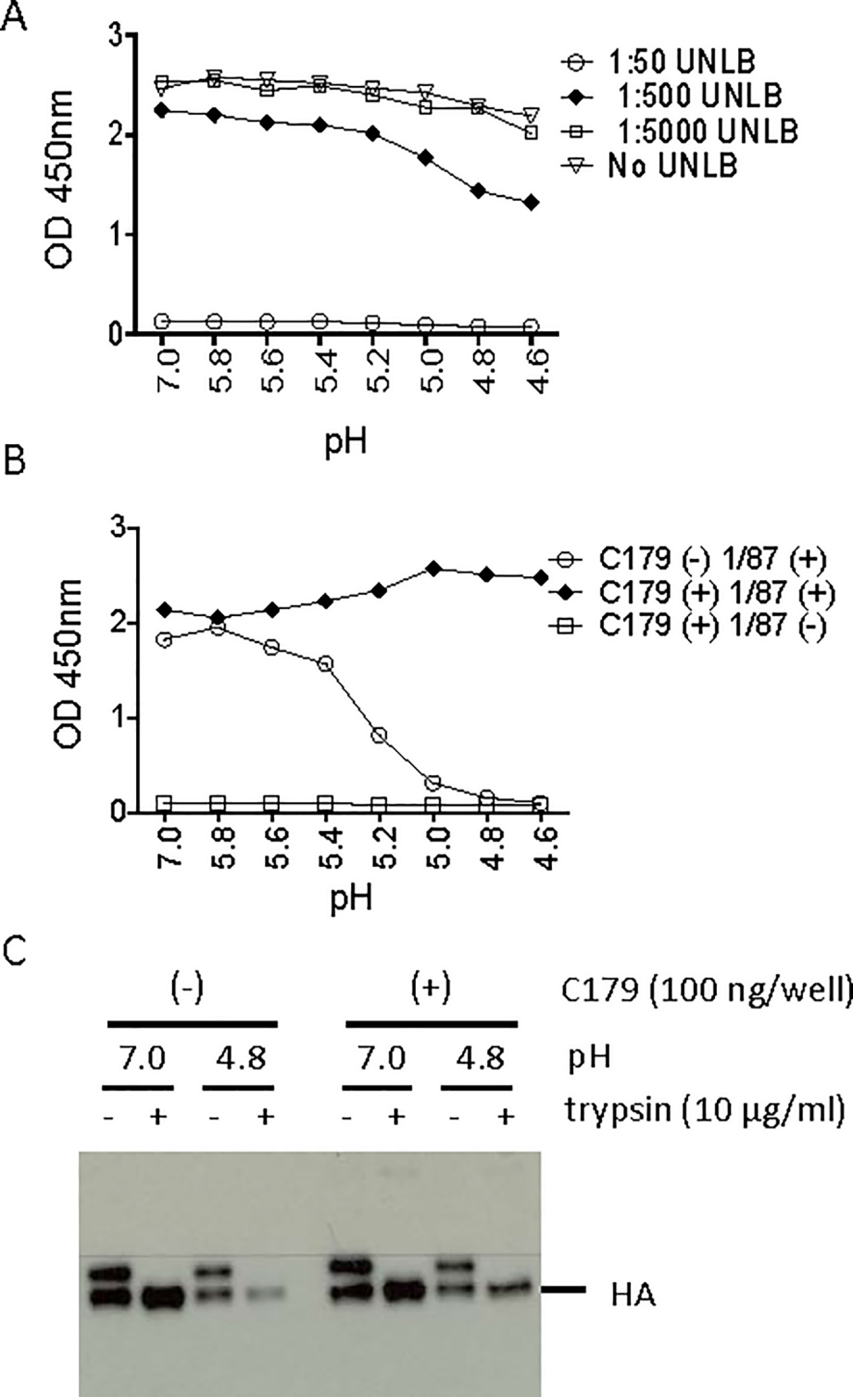
Inhibition of H2 rHA low pH induced conformational change by mAb C179. **A.** 1:50 unlabeled goat anti-mouse IgG (UNLB, Southern Biotech, AL) completely masked C179 that bound to H2 rHA. Because both C179 and 1/87 are mouse mAbs, a blocking step was required for detection specificity. H2 rHA coated nickel plates were incubated with 100 ng/well of C179 for 1 hour followed by incubation with diluent only, 1:5000, 1:500, or 1:50 diluted UNLB for 1 hour. The effects of this blocking step was confirmed by ELISA using HRP-conjugated goat anti mouse IgG (SouthernBiotech, AL). **B.** H2 rHA was bound to a nickel-coated 96-well plate, treated with 200 ng/ml trypsin, then incubated with or without mAb C179 (100 ng/well), and treated with pH adjusted buffer in 0.2 unit increments ranging from pH 4.6–5.8 and pH 7.0. The plate was washed once with PBS followed by blocking with UNLB (1:50), H2 rHA was fixed with 0.05% glutaraldehyde/PBS and washed. To ascertain the conformation of H2 rHA, the plates were incubated with the pH specific mAb 1/87 followed by incubation with an HRP-conjugated goat anti-mouse IgG. Reactions were terminated and the OD_450 nm_ was measured in ELISA. **C.** To confirm the results in Fig 3B, the protease susceptibility assay was performed without the blocking step. H2 rHA was bound to a nickel-coated 96-well plate, cleaved with 200 ng/ml of trypsin, incubated with or without mAb C179 (100 ng/well), treated with neutral (7.0) or low pH (4.8) buffer, and subsequently treated with trypsin at 0 or 10 μg/ml. Samples including both the released digested rHA and rHA remaining on the plate, were eluted from the nickel-coated plate by adding an equal volume of 2X non-reducing SLB supplemented with 1M imidazole, and were separated by SDS-PAGE under non-reducing conditions. PAGE-separated proteins were transferred to a nitrocellulose membrane and probed with an anti H2 HA mAb (2/9). HA proteins were detected by chemiluminescence with an HRP-conjugated secondary antibody.

To validate our HCCIA, we used the well-characterized bn mAb, C179, and the anti-H2 mAb 1/87, which recognizes the neutral pH conformation of H2 HA. Briefly, H2 rHA was immobilized on a nickel-coated 96-well plate, treated with exogenous trypsin, incubated in the presence or absence of C179, and subsequently exposed to pH adjusted buffer ranging from 4.6–5.8 and 7.0. To eliminate signal from HRP-conjugated goat anti mouse IgG binding to mouse mAb C179, an extra blocking step was performed instead of using HRP labeled or biotinylated mAb 1/87 in HCCIA in this study. As shown in Fig 3A, pre-treated plates with 1:50 diluted unlabeled goat anti-mouse IgG (UNLB, Southern Biotech, AL) completely blocked HRP-conjugated goat anti-mouse IgG binding to C179. After blocking, plates were fixed using glutaraldehyde and assayed for reactivity to anti H2 mAb, 1/87, specific to the neutral conformation of HA. As shown in Fig 3B, in the absence of C179 the anti-H2 mAb, 1/87, loses reactivity with the H2 rHA samples that were incubated with lower pH buffers, similar to that observed with mAbs HC31 and HC67 for H3 rHA (Fig 2C). Conversely, in the presence of 100 ng/well C179, the H2 rHA maintained reactivity with the 1/87 mAb, 100% OD_450nm_ value at pH 4.6 compared to that at pH 7.0 (Fig 3B), indicating that the C179 bn mAb was able to inhibit low pH induced HA conformational changes. In the complementary protease susceptibility assay, H2 rHA was shown to be resistant to further trypsin digestion (10 μg/ml) at pH 7.0 regardless of whether 100 ng/well of C179 is present or not (Fig 3C). On the other hand, only about 60% rHAs remained following pH 4.8 treatment compared to that at pH 7.0 treated samples as shown in Fig 3C, and rHAs were more protected from further trypsin digestion at 10 μg/ml trypsin concentration in the presence of 100 ng/well of C179 (Fig 3C). Thus, inhibition of H2 rHA low pH induced conformational change by C179 was confirmed with HCCIA and the proteinase susceptibility assay. Taken together with the data in Fig 2 and Fig 3, these data validate the use of the HCCIA as a reliable assay to detect bn mAbs that inhibit low pH induced HA conformational changes associated with membrane fusion activity.

### Analysis of human serum samples by HCCIA and proteinase susceptibility assays

**Fig 4:**
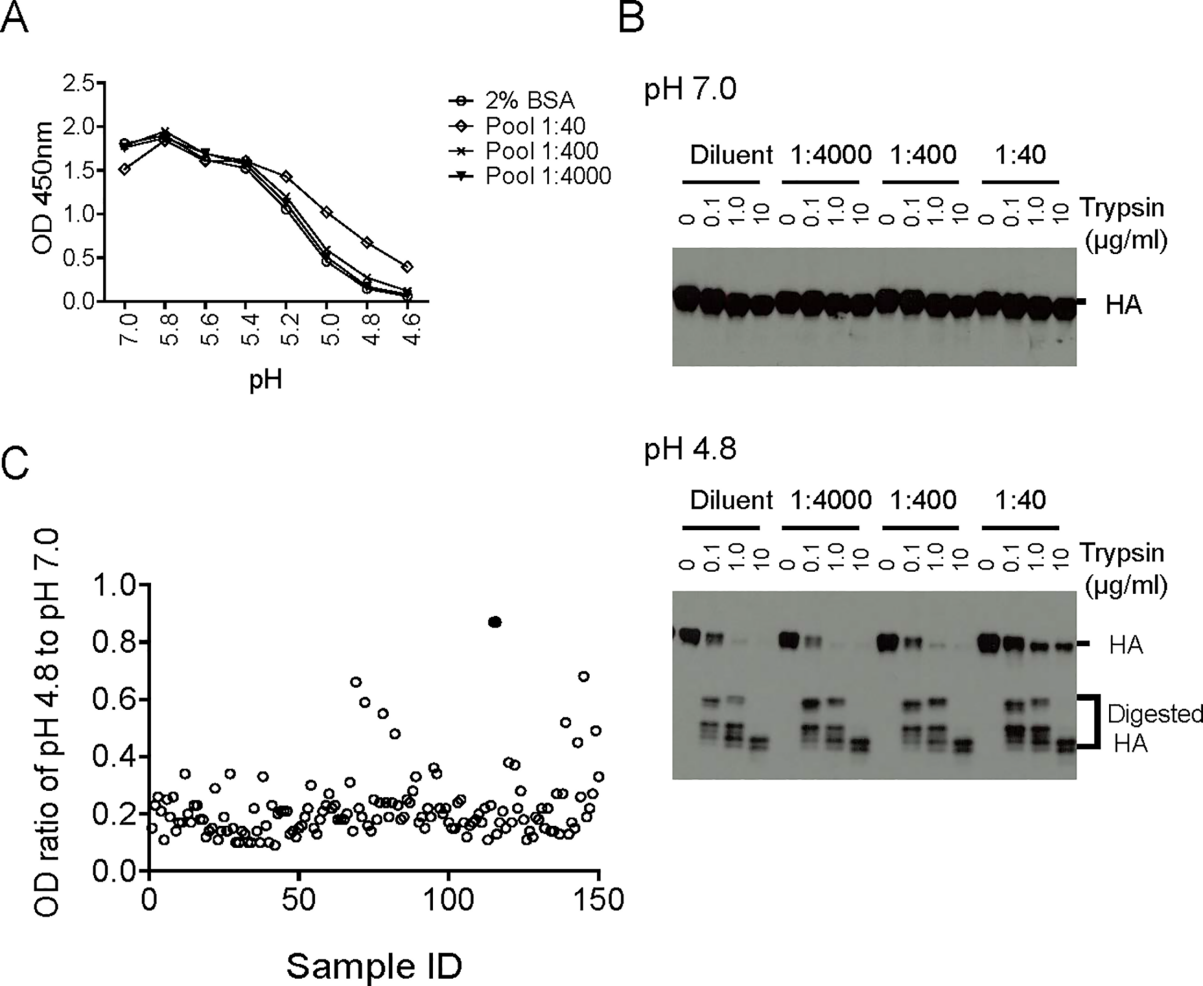
Low pH induced conformational changes of H3 rHA were inhibited by the human serum samples. **A.** Inhibition of low pH induced H3 rHA conformation change by a convenient human serum pool (Pool) in HCCIA. H3 rHAs bound to nickel-coated plates were treated with 100 μl of 200 ng/ml trypsin to cleave HA0 into HA1 and HA2. rHA coated plates were incubated with diluent only, 1:4000, 1:400, or 1:40 diluted Pool for 1 hour. The plate was washed and treated with a range of pH buffers followed by fixation with 0.05% glutaraldehyde/PBS. An ELISA was performed using a pH-specific mAb, HC31, and detected by measuring the OD at 450 nm. **B.** Inhibition of H3 rHA low pH induced conformation change by human serum pool in the proteinase susceptibility assay. The proteinase susceptibility assay was performed to confirm HA low pH conformational changes in Fig 4A. H3 rHAs bound to nickel-coated plates were treated with 100 μl of 200 ng/ml trypsin to cleave HA0 into HA1 and HA2. The rHA coated plate was incubated with either diluent only, 1:4000, 1:400, or 1:40 diluted Pool for 1 hour followed by treatment with pH 7.0 or pH 4.8 buffer. The rHAs were digested with 0, 0.1, 1, or 10 μg/ml trypsin, the samples including digestion mixture and rHA left on plate were eluted from the nickel-coated plate by adding an equal volume of 2X non-reducing SLB supplemented with 1M imidazole and were separated by SDS-PAGE under non-reducing conditions. PAGE-separated proteins were transferred to a nitrocellulose membrane and probed with rabbit anti A/Aichi/2/68 (H3N2) antisera. HA proteins were detected by chemiluminescence with an HRP-conjugated secondary antibody. **C.** Detection of the CCI against H3 rHA in normal human sera in HCCIA. In total, 150 normal human sera collected from US residents were tested at 1:400 dilution by HCCIA as described in the Fig 4A legend. The OD ratio of HC31 at pH 4.8 to pH 7.0 was plotted; the highest ratio positive sample, #115, highlighted as a filled circle.

The ultimate goal of this study was to develop a method to evaluate whether CCI (or fusion inhibition antibodies), which are potential bn Abs, could be stimulated by vaccination or natural infection. To examine whether this was feasible using HCCIA to achieve this aim, we performed serial experiments by using a convenient human serum pool (Pool) and further evaluated whether the inhibition of low pH induced HA conformational changes by antibodies could be detected. As shown in Fig 4A, when we tested various dilutions of the human serum pool, a slightly higher HC31 binding activity to H3 rHA at low pH was observed at the 1:40 serum dilution compared to a higher serum dilutions or diluent only control, indicating that the low pH induced conformational changes of H3 rHA were partially inhibited by the human serum pool. This modest titer is likely due to the relatively low concentration of CCI that are elicited following previous vaccination or natural infection. The HCCIA data was further confirmed by the protease susceptibility assay, which showed that cleaved H3 rHA incubated in pH 7.0 buffer was resistant to further trypsin digestion, whereas cleaved H3 rHA incubated at pH 4.8 was sensitive to trypsin digestion in a concentration-dependent manner when incubated with serum prior to low pH treatment (Fig 4B). These data suggest that antibodies in human serum samples that can inhibit low pH induced HA conformational changes were measurably detectable by HCCIA and the protease susceptibility assay beginning at a serum dilution of 1:40.

Next, we tested one hundred and fifty 1:400 diluted human serum samples collected from U.S. residents by HCCIA using H3 rHA at pH 7.0 and pH 4.8. Some human sera compete the binding sites for HC3 and HC31 at 1:40 dilution, fortunately, OD_450nm_ at pH 7.0 were higher than 0.8 at 1:400 dilution for all tested serum samples. The OD_450nm_ ratios of mAb HC31 at pH 4.8 to pH 7.0 ranged from 0.87 to 0.09 (Fig 4C) while the ratio of human serum pool was about 0.15 (Fig 4A), indicating the presence of various level of CCI in these human serum samples.

### Demonstration of neutralizing activity from functional stalk-binding antibodies after removal of HA head-binding antibodies

**Fig 5:**
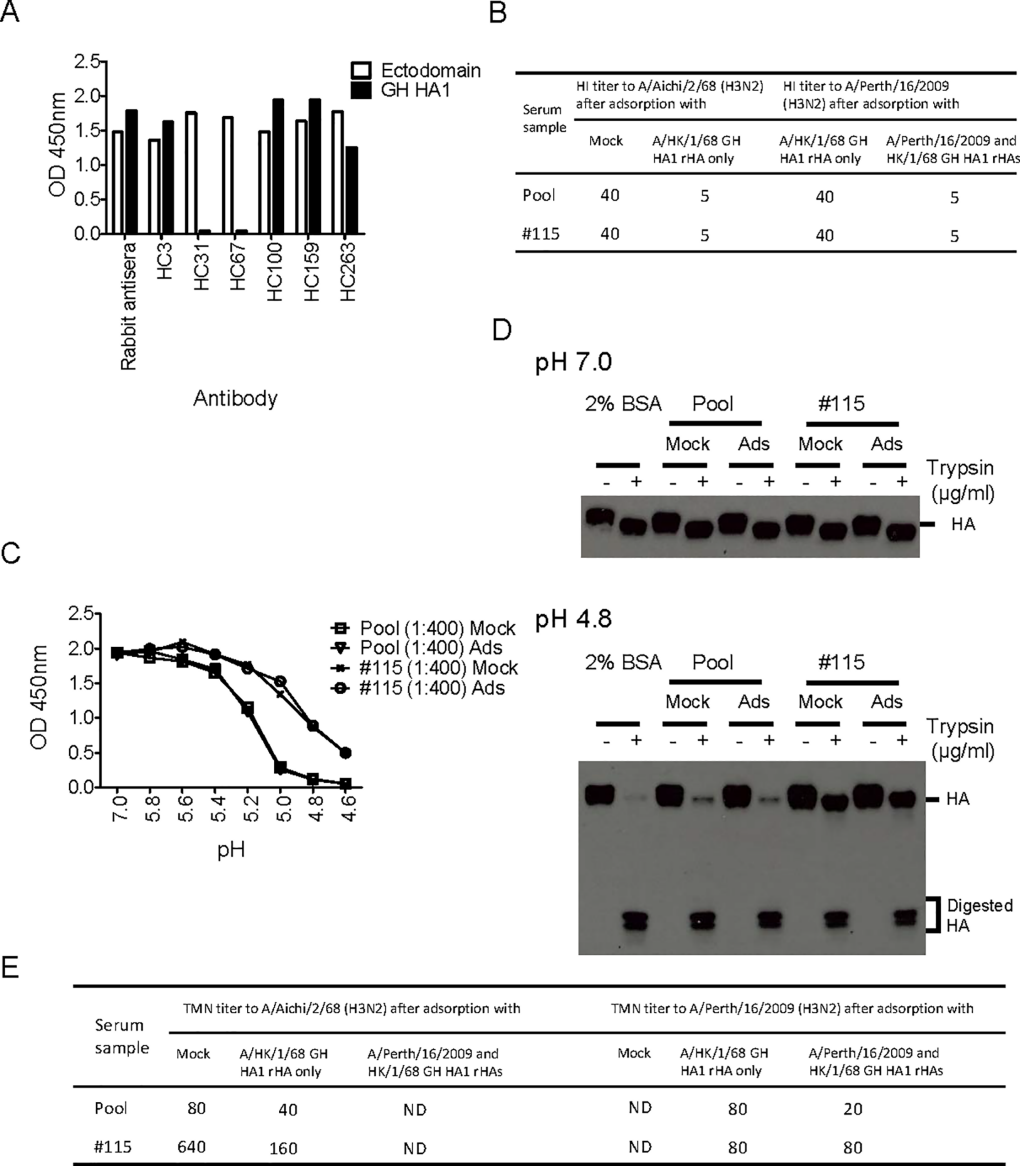
CCI showed virus neutralizing activity in TMN after removal of head-binding antibodies. **A.** Correct folding of major epitopes for GH HA1 from A/Hong Kong/1/68 (H3N2) (Sino Biological, Inc. China) was confirmed by appropriate Ab binding profiles. GH HA1 from A/Hong Kong/1/68 (H3N2) and ectodomain H3 rHA from A/Aichi/1/68 (H3N2) were coated on a nickel-coated plate, an ELISA was performed by using rabbit antisera and a panel of conformation specific mAbs. **B.** Removal of HI antibodies by serum adsorption with GH HA1 rHAs. The convenient human serum pool (Pool) and #115, the highest positive sample in HCCIA, were adsorbed with GH HA1 from A/Hong Kong/1/68 (H3N2) or double adsorbed with GH HA1 proteins from A/Hong Kong/1/68 (H3N2) and A/Perth/16/2009 (H3N2). Hemagglutination inhibition assays were performed by using A/Aichi/2/68 and A/Perth/16/2009. CCI were consistently present after mock or serum adsorption with GH HA1 in HCCIA **(C)** and the proteinase susceptibility assay **(D). E.** CCI neutralized A/Aichi/2/68 (H3N2) and A/Perth/16/2009 after head-binding antibodies were removed by serum adsorption with GH HA1 rHAs. ND: not done.

The majority of neutralizing antibodies elicited upon vaccination or natural infection are hemagglutination inhibition (HI) antibodies that bind to antigenic sites in the HA head domain near the receptor binding site [4]. To reduce the presence of head-binding neutralizing Abs and their effects, we used a serum adsorption assay to remove HA head-binding Abs and investigated whether the remaining antibodies (mainly stalk-binding antibodies) in the serum sample inhibited the low pH induced HA conformational changes associated with membrane fusion activity. First, we evaluated the folding of GH HA1 from A/Hong Kong/1/68 (Sino Biological, Inc. China) by ELISA. As shown in Fig 5A, most conformational epitope specific mAbs reacted with the ectodomain of rHA and GH HA1 at similar levels, with the exception of HC31 and HC67, which was expected because these mAbs bind to the trimeric HA head domain at neutral pH conformation. Thus, we concluded that most conformational epitopes of GH HA1 are folded correctly. We also used another GH HA1 from A/Perth/16/2009 that was not evaluated for proper folding due to the lack of well-characterized mAbs.

The human serum pool (Pool) and the highest positive sample in HCCIA, #115 (Fig 4C), were adsorbed by GH HA1 rHA from A/Hong Kong/1/68 only or adsorbed with both GH HA1 rHAs from A/Hong Kong/1/68 and A/Perth/16/2009 to remove HA head-binding Abs. As expected, total GH HA1 rHA-binding antibodies including HI Abs were removed by adsorption with GH HA1 as shown in Fig 5B and S2 Fig. Next, both HCCIA and protease susceptibility assays were performed using mock adsorbed and GH HA1-adsorbed sera to demonstrate the stalk-binding Abs remain intact in the adsorbed serum sample. As shown in Fig 5C, sample #115 showed greater inhibition of low pH induced HA conformational changes compared to the human serum pool at a 1:400 dilution, and the ability of the mock or GH HA1 adsorbed samples to inhibit low pH induced conformational changes of the rHA was indistinguishable (Fig 5C). This observation was further confirmed using the protease susceptibility assay where low pH treated HAs were protected equally for both mock or GH HA1 adsorbed #115 serum samples (Fig 5D). The neutralizing activities of these serum samples after mock or GH HA1 adsorption were analyzed using A/Aichi/2/68 (H3N2) and A/Perth/16/2009 (H3N2) viruses. The neutralization titers against A/Aichi/2/68 in TMN were reduced with GH HA1-adsorbed serum from A/Hong Kong/1/68 for both samples, as expected. GH HA1 adsorbed Pool also showed reduced neutralization titers against both A/Aichi/2/68 and A/Perth/16/2009 (Fig 5E). Interestingly, the neutralization titers in TMN of sample #115 against A/Perth/16/2009 remained at similar levels after adsorption with GH HA1 rHA from A/Hong Kong/1/68 only and double adsorption with GH HA1 rHAs from A/Hong Kong/1/68 and A/Perth/16/2009. Collectively, these results demonstrated that Abs in these serum samples inhibit low pH induced conformational changes of H3 rHA which could neutralize both A/Aichi/2/68 and A/Perth/16/2009 viruses following removal of head-binding Abs (Fig 5E).

## Discussion

The primary focus of this study was to develop a reliable assay for detecting HA stalk-binding CCI that are potentially broadly neutralizing. We have described the development of an HCCIA and a complementary protease susceptibility assay, both of which are capable of being scaled for high throughput. Of note, both assays can be performed under BSL-2 conditions, effectively increasing their utility. Both assays were validated using recombinant H2 and H3 HAs, which are representative of group 1 and group 2 HAs and have well-characterized conformation specific monoclonal antibodies available. Of particular importance, we have shown a proof-of-concept detection of the highest HCCIA ratio sample #115 that inhibits low pH induced HA conformational changes associated with membrane fusion activity (Fig 4C). Further, we have shown that, by removing HA head-binding Abs, we were able to specifically enhance the detection of these stalk-binding antibodies that are potentially broadly neutralizing.

The cleavage of HA0 into disulfide-linked HA1 and HA2 to generate the proteolytically active metastable conformation of HA capable of undergoing low pH induced conformational change is essential for influenza virus infectivity [46, 55]. Because the starting material used in this study was trimeric HA0, it was necessary to treat the rHAs with trypsin; however, based on initial studies, we found that it was prudent to generate rHA constructs without an authentic thrombin cleavage site to reduce protein loss during the trypsin cleavage of HA0 (S1 Fig). We utilized well-characterized conformation-specific Abs to verify that the rHAs were behaving as expected and that both assays were measuring the presence or absence of CCI. For both rHAs, we confirmed that in the absence of CCI, they lost reactivity to neutral pH conformation-specific detection mAbs (Figs 2C, 3B, 4A and 5C) and became susceptible to further digestion by trypsin at low pH (Figs 2D, 3C, 4B and 5D).

The principle of HCCIA was confirmed using bn mAb C179 and reproducibly reflected results obtained with the conventional proteinase susceptibility assay (Figs 2D and 3C), which were consistent with previous studies [21, 26, 37, 38]. These results show that bn mAbs can be analyzed by HCCIA, a convenient ELISA-based assay, which allows for high throughput, particularly since histidine-tagged rHAs on a nickel-coated plate are oriented as would be on the viral surface and can be easily eluted by imidazole for use in a conventional proteinase susceptibility assay (Figs 2D, 3C, 4B and 5D). This 96-well nickel-coated plate based HCCIA and improved protease susceptibility assay are applicable for evaluating human or animal antibodies that can inhibit low pH induced HA conformational changes that are potentially broadly neutralizing. Of note, either a blocking step or labeling pH specific antibodies is necessary when the bn mAbs from the same species that are used to generate detection mAbs (such as 1/87) as shown in Fig 3.

A disadvantage of using nickel-coated plates is the possible interruption of nickel-histidine interactions by low pH treatment (pH 6.0-pH 4.0) [56] or the potential detrimerization of the foldon sequence at low pH (pH 4.3-pH 1.6) [57]. Interestingly, the results demonstrated that the protein loss from low pH treatment did not affect HCCIA (Figs 2C and 3B), because 50–100 ng/well of rHA could achieve maximum OD_450 nm_ in ELISA and the excess rHA was used in this study (200 ng/well) (S3 Fig). On the other hand, 20–40% rHA loss due to low pH (pH 4.8) treatment was observed in the proteinase susceptibility assay (Figs 2D, 3C, 4B and 5D). Fortunately, the principle of the conventional proteinase susceptibility assay was to compare rHA amounts following digestion with or without 10 μg/ml trypsin at either pH 7.0 or pH 4.8 treatment. Furthermore, the conventional proteinase susceptibility assay was used to confirm our results in HCCIA in this study. It will not be necessary when the HCCIA becomes a widely accepted assay to evaluate CCI. Low pH conformational changes of H3 rHA was confirmed by using regular high binding ELISA plates (S4 Fig), currently we are developing HCCIA for use on regular high binding ELISA plates to avoid the potential interference of low pH in nickel-histidine interactions. The streptavidin binding peptide tagged rHA could be an interesting approach for HCCIA, since the interaction is not pH dependent and rHA could be eluted using biotin [58].

For the detection of potential bnAbs in human serum samples by HCCIA using mouse mAbs as the detection antibodies (such as HC31, HC67, and 1/87), we confirmed the absence of cross-reactivity of HRP-goat anti mouse IgG antisera (Southern Biotech, AL) with human IgG (S5 Fig). The convenient human serum pool showed a dilution-dependent inhibition of low pH conformational changes of H3 rHA by HCCIA and the proteinase susceptibility assay (Fig 4A and 4B), though it is only detectable at the 1:40 dilution. The presence of CCI against H3 rHA in 150 normal human serum samples at a 1:400 dilution was confirmed by HCCIA using mAb (HC31). The OD ratios of mAb HC31 at pH 4.8 to pH 7.0 ranged from 0.87 (serum sample #115 showed in filled circle) to 0.09 (Fig 4C). Antibodies that inhibited the low pH induced HA conformational changes, would inhibit HA membrane fusion activity and are potentially broadly neutralizing.

Several HA head-binding monoclonal antibodies have been shown to block low pH induced HA conformational changes [13, 16, 17, 59], suggesting such antibodies may be present in human sera and will complicate the interpretation of the results of HCCIA and proteinase susceptibility assays. Therefore, we introduced a GH HA1 rHA-coated nickel bead adsorption step to remove HA head-binding antibodies from the human serum samples to specifically detect stalk-binding antibodies. Initially, the conformation of GH HA1 was confirmed using an anti-H3 HA mAb panel used in previous studies [46, 47]. As shown in Fig 5A, the major epitopes of monomeric GH HA1 from A/Hong Kong/1/68 fold correctly; furthermore, HI antibodies were adsorbed completely (Fig 5B), as expected GH HA1 did not bind to HC31 and HC67 antibodies which recognize trimeric epitopes (Fig 5A). After head-binding antibodies were removed via adsorption (Fig 5B and S2 Fig), stalk-binding antibodies in adsorbed sera still inhibited low pH induced HA conformational changes, which was confirmed by the HCCIA and proteinase susceptibility assays (Fig 5C and 5D). Interestingly, stalk-binding antibodies in sample #115 showed neutralizing activities against both A/Aichi/2/68 and A/Perth/16/2009 without HI antibodies (Fig 5B and 5E). These results suggest that HCCIA can detect CCI in human serum samples that are potentially broadly neutralizing.

It is not always appropriate to correlate *in vivo* and *in vitro* neutralizing activities, since both neutralizing and non-neutralizing antibodies can be protective *in vivo* [15, 60]. Both HA head-binding and HA stalk-binding broadly neutralizing antibodies are involved in various neutralizing mechanisms including blocking virus binding to host cells, blocking virus release, inhibiting low pH induced HA conformational changes, and blocking cleavage of HA0 [3, 13–17, 20]. Additionally, Fc-receptors or complement-related mechanisms are involved in *in vivo* protection, such as antibody-dependent cell mediated cytotoxicity (ADCC), antibody-dependent cellular phagocytosis (ADCP), antibody-dependent respiratory burst (ADRB) activity, or complement-dependent cytotoxicity (CDC) [14, 15], as well as T cell responses[60]. Epitopes of HA are located in both HA1 and HA2 [61], there are no evidences that all HA stalk-binding antibodies show HA low pH conformational change inhibition or broadly neutralizing activities. Several studies also showed that broadly neutralizing anti HA head and anti HA stalk mAbs require both Fc-FcγR interactions and HA RBS-sialic acid on effector cells for optimal ADCC activation and *in vivo* protection [16, 62, 63].

Initial optimization of C179 concentration in HCCIA showed that 25–100 ng/well of C179 could protect 100% of 200 ng/well H2 rHA (S6 Fig). Further studies are needed to optimize the assay as well as increase the range of human serum samples such as those from universal vaccine studies; additional samples will help to define the threshold of OD ratio that correlates with protection.

The HCCIA is dependent on pH-specific detection mAbs (such as HC31, HC67, and 1/87), the recent development of techniques [21–23] could be utilized to screen more mAbs that are potentially pH-specific. Some pH-specific detection mAbs against other HA subtypes in either group 1 or group 2 were identified and under further characterization. In general, it has been suggested that lower numbers of bnAbs against group 2 HAs are found due to an additional glycosylation site at HA1 residue 38 [26]. We are currently utilizing HCCIA to detect potential bnAbs against group 1 and other group 2 rHAs.

Currently, various approaches are being utilized to elicit functional stalk-binding bnAbs and to design and develop HA stalk structure-based vaccines, some of which are being examined in pre-clinical and clinical trials [1, 5, 9, 35], but there is no validated high-throughput assay that can directly detect CCI in human or animal serum samples. We have shown that HCCIA and the complementary proteinase susceptibility assay can be used to detect stalk-binding Abs that inhibit low pH induced HA conformational changes associated with membrane fusion, and may be scaled up to accommodate high-throughput use. Additionally by removing competing HA head-binding Abs, serum samples can be improved for specific detection of stalk-binding Abs.

We suggest that these assays will be valuable additions to the available tools for measuring CCI that inhibit fusion activity targeted by HA stalk targeting vaccine strategies and therapies designed to protect against a broad spectrum of HA subtypes.

## Supporting information

S1 Fig Deleted thrombin cleavage site (ΔTCS) H3 rHA was used in this study. The trypsin digestion mixture or samples from eluted rHAs bound to nickel-coated plates were tested by Western blot by using rabbit antisera. **A.** Less ΔTCS rHAs were detected in trypsin digestion mixture than rHA with thrombin cleavage site (TCS). **B.** More ΔTCS rHA bound to nickel-coated plate than rHA with thrombin cleavage site.
(TIFF)

S2 Fig Antibodies were removed by serum adsorption with either HK/1/68 GH HA1 rHA only (Ads- HK only) or both HK/1/68 and Perth/16/2009 GH HA1 rHAs (Ads- HK/Perth). The plate was coated with A/HK/1/68 GH HA1 rHA (120 ng/well) (A and C) or A/Perth/16/2009 GH HA1 rHA (120 ng/well) (B and D), probed with 2-fold diluted Pool (A and B) or #115 (C and D) starting from 1:800 in ELISA.
(TIFF)

S3 Fig H3 rHA amount and OD 450nm. (TIFF)

S4 Fig Low pH conformational change of H3 rHA on 2HB 96-well ELISA plate. (TIFF)

S5 Fig HRP-conjugated Goat anti mouse IgG (SB) (HRP-GAM IgG) does not cross-react with human IgG standard (200 ng/well) at 1:2000 working dilution. The plate was coated with human IgG standard (200 ng/well) that was detected by HRP-conjugated goat anti human IgG (HRP-GAH IgG), but was not by HRP-GAM IgG.
(TIFF)

S6 Fig Inhibition of H2 rHA (200 ng/well) low pH conformational change by C179. (TIFF)
