# Supplementary figures and images for: Development of a high-throughput assay to detect antibody inhibition of low pH induced conformational changes of influenza virus hemagglutinin
Source: PLoS One. 2018 Jun 27;13(6):e0199683. doi: 10.1371/journal.pone.0199683 (PMC6021090; doi:10.1371/journal.pone.0199683)

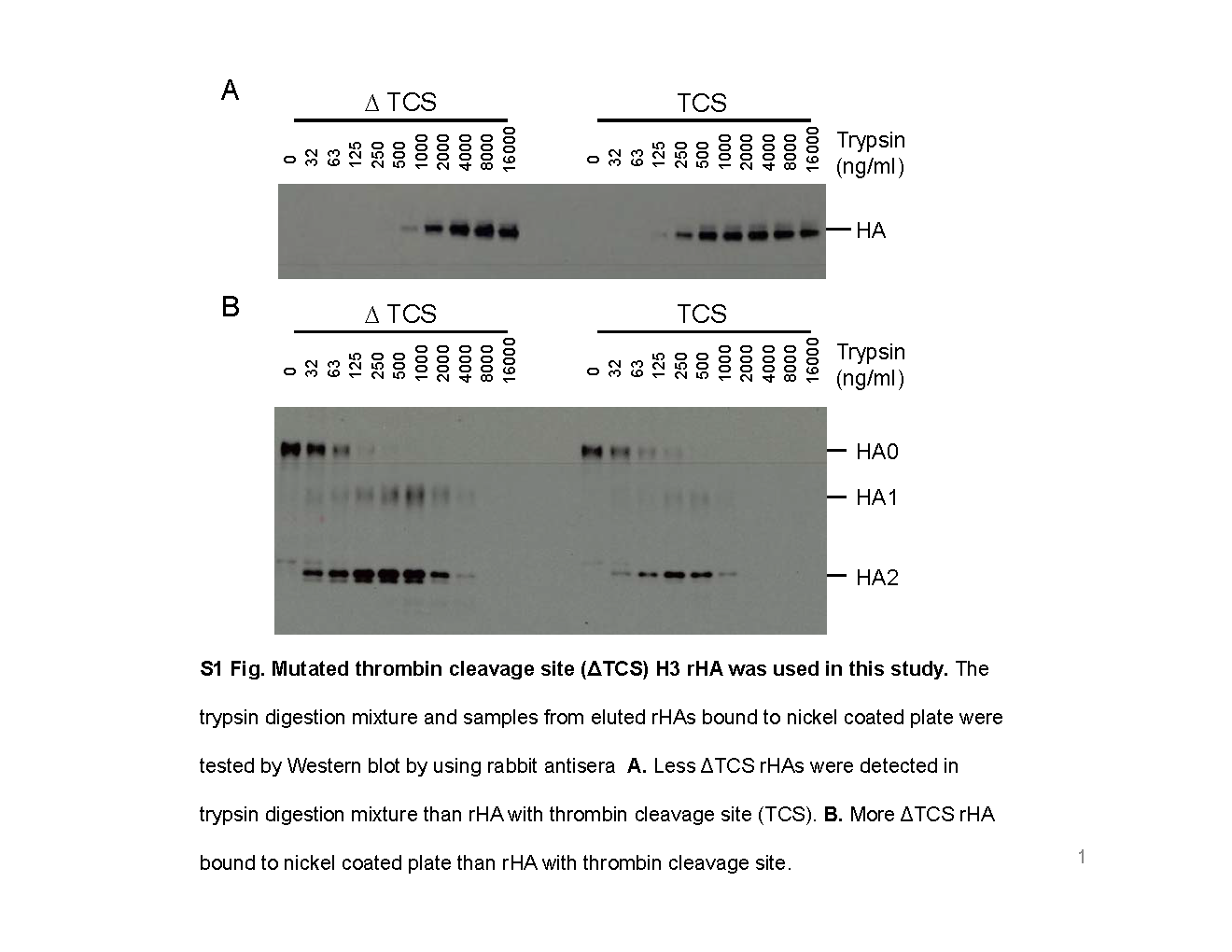

Supplement: S1 Fig — The trypsin digestion mixture or samples from eluted rHAs bound to nickel-coated plates were tested by Western blot by using rabbit antisera. A. Less ΔTCS rHAs were detected in trypsin digestion mixture than rHA with thrombin cleavage site (TCS). B. More ΔTCS rHA bound to nickel-coated plate than rHA with thrombin cleavage site. (TIFF) [file pone.0199683.s001.tiff]

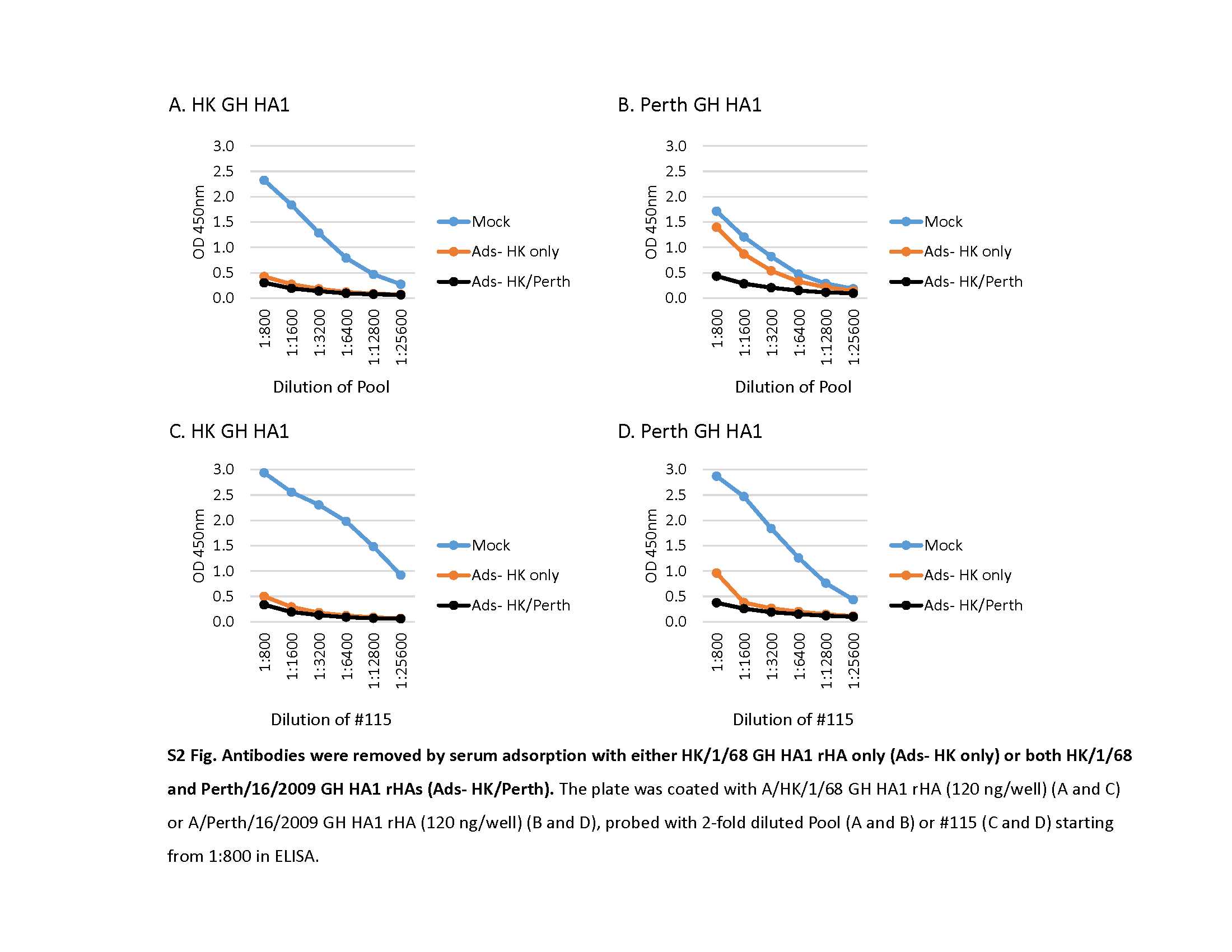

Supplement: S2 Fig — The plate was coated with A/HK/1/68 GH HA1 rHA (120 ng/well) (A and C) or A/Perth/16/2009 GH HA1 rHA (120 ng/well) (B and D), probed with 2-fold diluted Pool (A and B) or #115 (C and D) starting from 1:800 in ELISA. (TIFF) [file pone.0199683.s002.tiff]

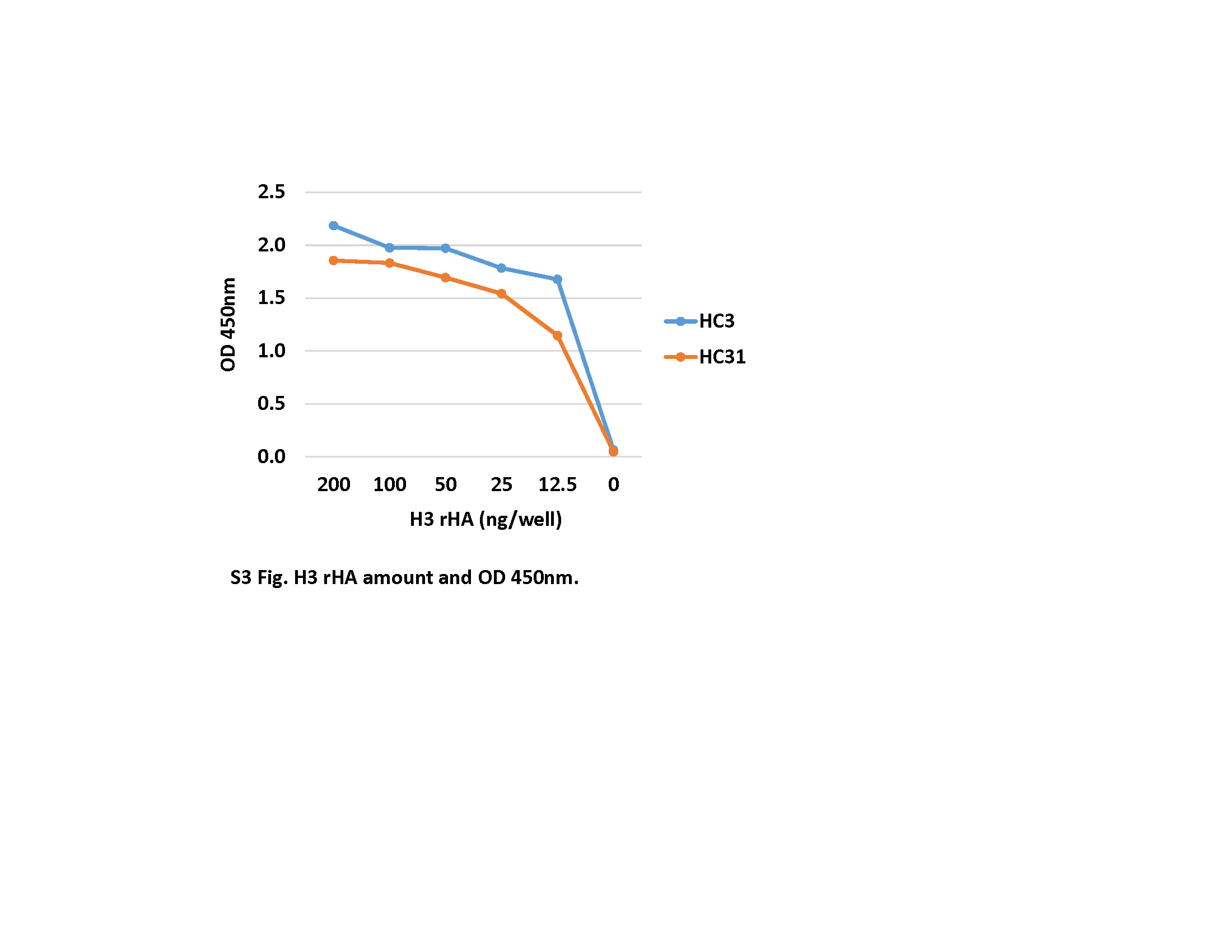

Supplement: S3 Fig — (TIFF) [file pone.0199683.s003.tiff]

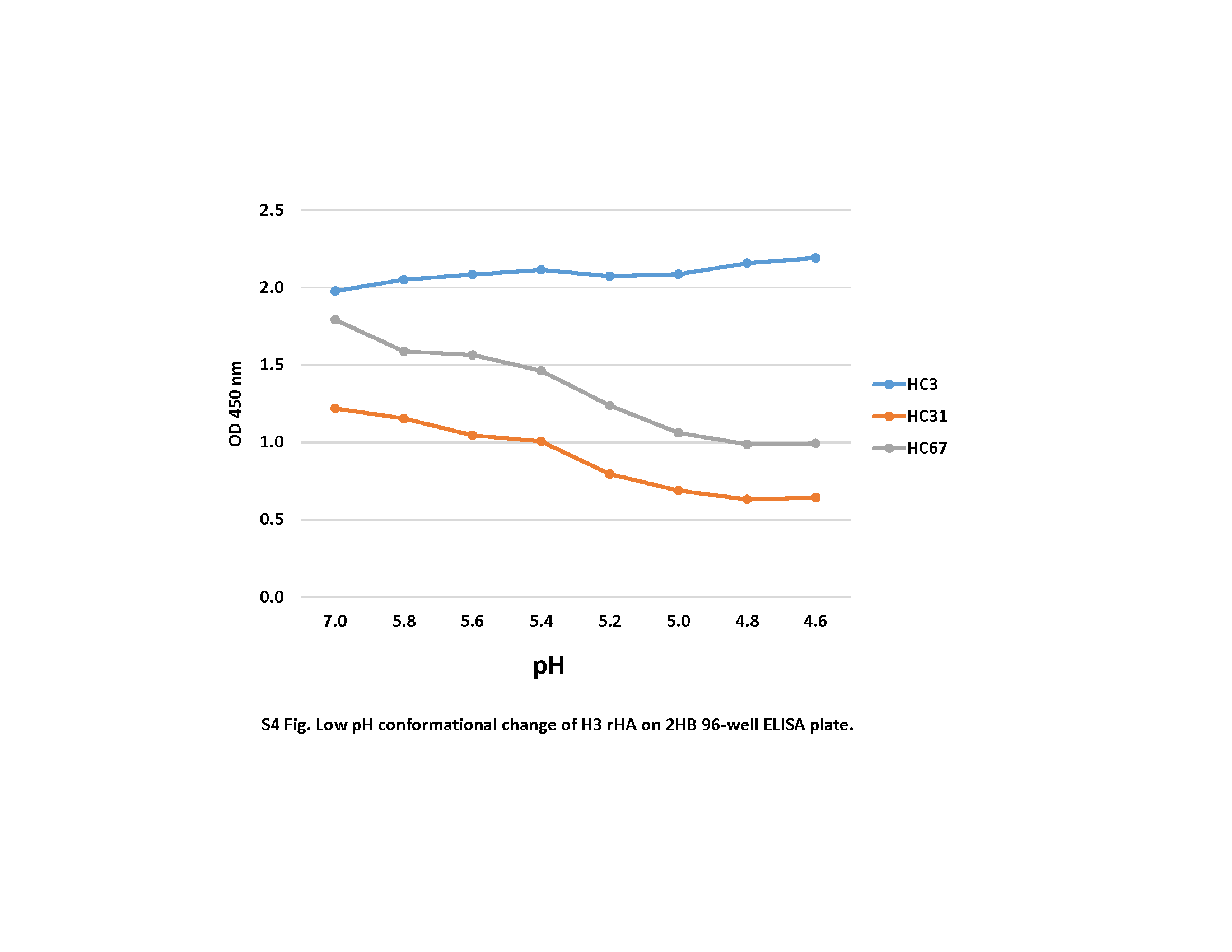

Supplement: S4 Fig — (TIFF) [file pone.0199683.s004.tiff]

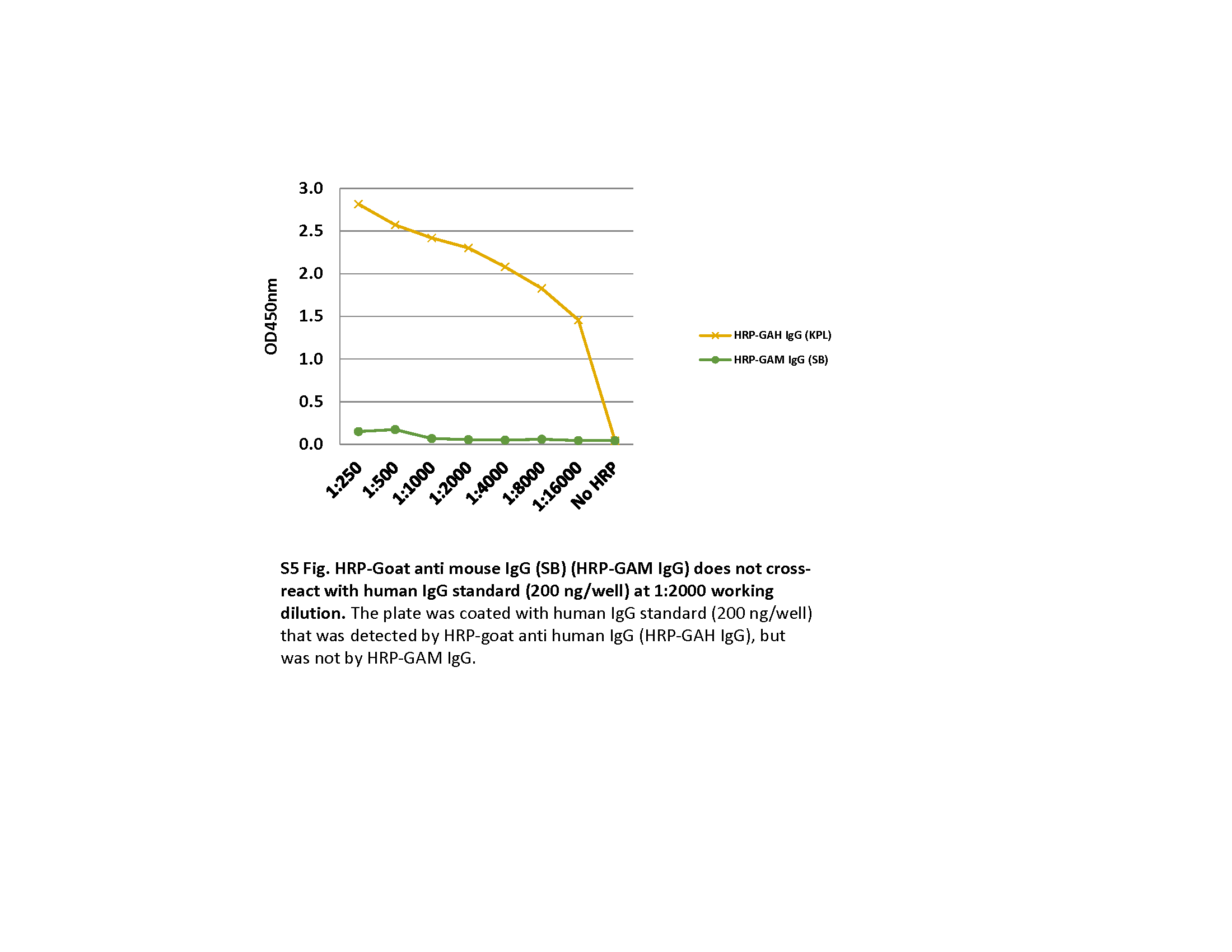

Supplement: S5 Fig — The plate was coated with human IgG standard (200 ng/well) that was detected by HRP-conjugated goat anti human IgG (HRP-GAH IgG), but was not by HRP-GAM IgG. (TIFF) [file pone.0199683.s005.tiff]

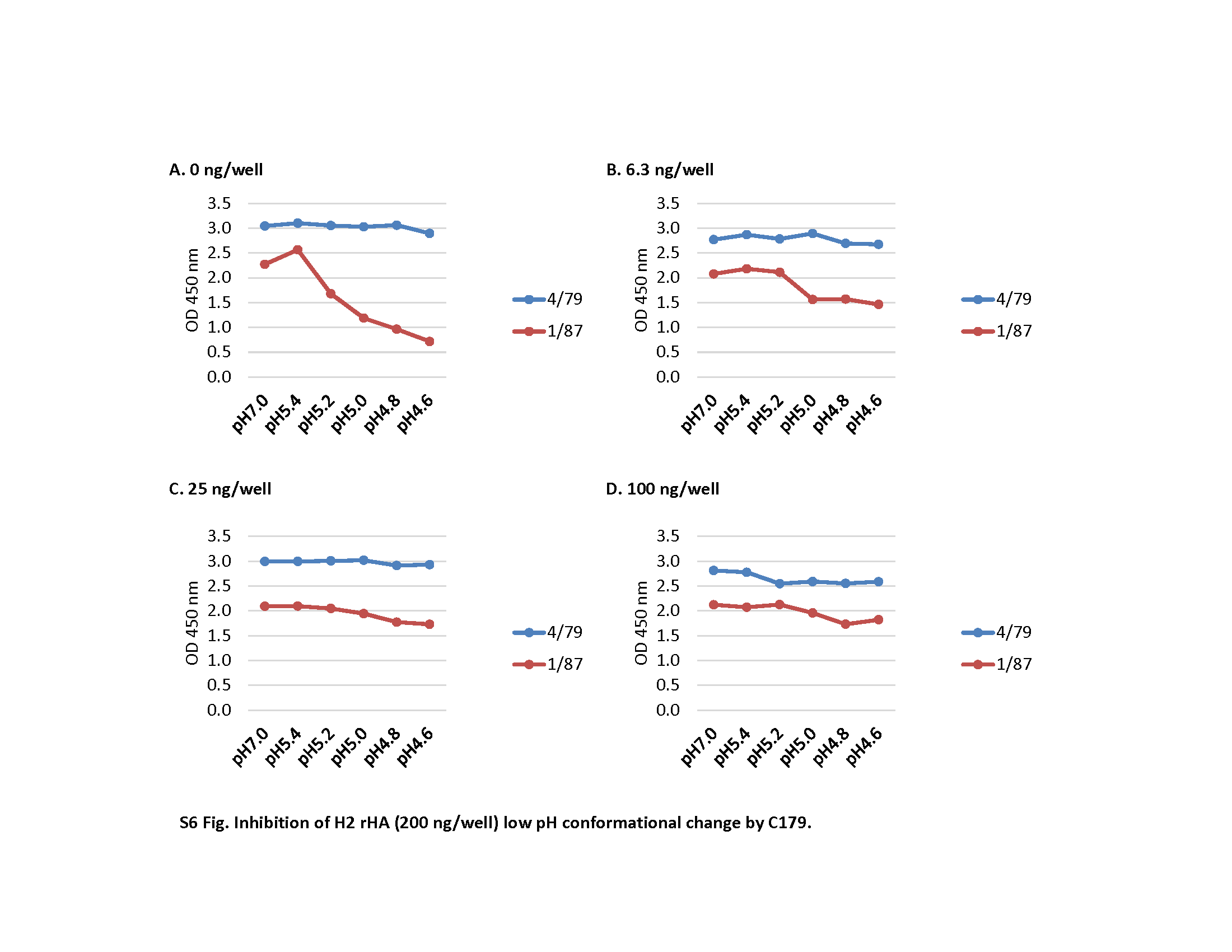

Supplement: S6 Fig — (TIFF) [file pone.0199683.s006.tiff]
